# Appreciating doubts about HIV medicine

**DOI:** 10.7448/IAS.18.1.20717

**Published:** 2015-12-08

**Authors:** Christy E Newman

**Affiliations:** Centre for Social Research in Health, UNSW Australia, Sydney, Australia

Taking medicines for a range of ills is normalized in most societies with supported access to health care, yet many people remain reluctant to use recommended medications. We know from the social research on immunization that little is gained by marginalizing or shaming those with hesitations or fears about health directives, particularly since non-compliance can often be explained as much by the complexities of access – even in high-income settings – as by consumer doubts in the legitimacy of conventional biomedicine.

Yet, within the increasing global focus on early and lifelong use of antiretroviral therapies (ART), there appears to be reduced opportunities to express consumer concerns or fears in a supportive context, particularly given the broader public health benefits now recognized for HIV treatment. This opens up important questions about how government and advocacy organizations can keep conversations about treatment use open and supportive, recognizing that consumers have rights to both access these lifesaving and prevention medicines and to hold doubts about them.

It is undoubtedly an exciting time in HIV medicine, with a growing range of benefits of ART now recognized for the health, relationships and inclusion of people with HIV around the world. However, relatively less attention has been paid to the shadow side of this “game-changing” development: the stories told less often, or less visibly, from the minority of those people living with HIV in high-income nations who are not currently using the recommended treatments, or indeed, those who are using them but feel conflicted and concerned, and risk using them incorrectly or discontinuing use over time.

Our qualitative social research with people with HIV in Australia who were not using ART at the time of interview reported a diverse range of well-considered reasons offered to explain this departure from the recommended norms [1–3]. Some had been recently diagnosed, and did not feel ready to make the commitment required when embarking on a lifelong therapeutical regimen requiring strict daily adherence. Others faced challenges in accessing therapies due to their immigration status, financial situation or geographic location. And a proportion were not able to use conventional treatments for other health reasons, or had prescribing doctors who remained cautious about recommending “early” treatment.

Some of our participants also provided reasons for non-use of treatment which revealed a deep distrust of medicine, yet this is by no means unique to HIV. Indeed, as a general observation, a view shared by all of our participants – likely to be recognized in most other areas of medicine – is that initiating a lifelong regimen of pharmaceutical medication requires careful thought and a deep and considered commitment. Modern health consumers are typically well aware that medications have the potential (no matter how small) to do harm as well as good, and that science cannot guarantee they will be protected from unintended effects in the short or long term. They also know that achieving perfect adherence is challenging for anyone, and in the case of HIV, failing to maintain a meticulous regimen of pill-taking risks the worrying development of drug-resistant HIV, and a subsequent reduction in the efficacy of treatment.

Adding a public health imperative into this complex mix does not always sit easily with those who hold doubts about medicine, even though the concept of treating for prevention benefit was generally well understood by our participants. As just one example from our research, “Sarah,” a single parent, believed she posed no risk of onward transmission in the way she was currently managing her HIV, and felt unmoved by messages suggesting she should start treatment for the public good. As she put it: “Hmm, protecting others from acquiring HIV? Not a good enough reason for me to start meds.” Sarah is not waiting for experts to come up with better reasons to convince her to override her concerns about starting treatment. For her, feeling unsure about HIV medications – their safety, purpose and value – is a good enough reason to refuse them, no matter what the weight of evidence suggests regarding their potential benefits; individual or public. Sarah does not share the more optimistic views of other people with HIV in Australia, many of whom are energetically embracing the many clinical and social benefits of treatment use. But even if her views do not represent the majority, Sarah's position deserves understanding, and certainly recognition that her doubts are unlikely to magically resolve without considered attention and empathic support.

The field of HIV medicine has a number of important questions to now debate regarding the mechanisms through which the communities who are the target of contemporary treatment and prevention technologies can be meaningfully engaged regarding the social complexities of treatment use and non-use. We need to ensure that the growing focus on treatment uptake avoids contributing to practices or perceptions of coercion, which risk forcing those with even minor doubts into stronger positions of treatment refusal and mistrust in the healthcare system. Just as there is much that the HIV sector can learn from other fields of public health in this regard, there is also potentially much to contribute back to those fields in return.

To that end, we need to be asking careful questions about how we can promote these medical interventions as genuinely remarkable, while also appreciating and validating the right of people with HIV to hold doubts. How can we avoid positioning those with concerns about the safety or trustworthiness of an evidence-based medical recommendation as irrational or irresponsible, while also recognizing that not all reasons to refuse medicine can or should be viewed as “equal”? And how can we facilitate opportunities for dialogue, in which people with alternative or minority views feel supported and respected, while also strongly advocating for the benefits of these lifesaving and transmission-preventing medicines?

The coming years will require thoughtful consideration regarding how to understand and respond to consumer doubts about treatments if we want all people with HIV to feel engaged and supported in the global response.

## Author information

Christy E Newman contributes to the HIV and sexual health team of the Centre for Social Research in Health at UNSW Australia, previously the National Centre in HIV Social Research, a leading global research institute in social aspects of HIV and related diseases.
